# Minimally invasive treatment of hepatic adenoma in special cases

**DOI:** 10.1590/S1679-45082013000400021

**Published:** 2013

**Authors:** Felipe Nasser, Breno Boueri Affonso, Francisco Leonardo Galastri, Bruno Calazans Odisio, Rodrigo Gobbo Garcia

**Affiliations:** 1 Hospital Israelita Albert Einstein, São Paulo, SP, Brazil; 2 MD Anderson Cancer Center, Houston, United States

**Keywords:** Adenoma, liver cells/therapy, Embolization, therapeutic, Liver/injuries, Case reports

## Abstract

Hepatocellular adenoma is a rare benign tumor that was increasingly diagnosed in the 1980s and 1990s. This increase has been attributed to the widespread use of oral hormonal contraceptives and the broader availability and advances of radiological tests. We report two cases of patients with large hepatic adenomas who were subjected to minimally invasive treatment using arterial embolization. One case underwent elective embolization due to the presence of multiple adenomas and recent bleeding in one of the nodules. The second case was a victim of blunt abdominal trauma with rupture of a hepatic adenoma and clinical signs of hemodynamic shock secondary to intra-abdominal hemorrhage, which required urgent treatment. The development of minimally invasive locoregional treatments, such as arterial embolization, introduced novel approaches for the treatment of individuals with hepatic adenoma. The mortality rate of emergency resection of ruptured hepatic adenomas varies from 5 to 10%, but this rate decreases to 1% when resection is elective. Arterial embolization of hepatic adenomas in the presence of bleeding is a subject of debate. This observation suggests a role for transarterial embolization in the treatment of ruptured and non-ruptured adenomas, which might reduce the indication for surgery in selected cases and decrease morbidity and mortality. Magnetic resonance imaging showed a reduction of the embolized lesions and significant avascular component 30 days after treatment in the two cases in this report. No novel lesions were observed, and a reduction in the embolized lesions was demonstrated upon radiological assessment at a 12-month follow-up examination.

## RESUMO

O adenoma hepatocelular é um tumor benigno raro, que apresentou aumento do número de diagnósticos nas décadas de 1980 e 1990, o que foi atribuído à difusão dos contraceptivos hormonais orais, e à melhor disponibilização e ao avanço dos exames radiológicos. Apresentamos aqui o relato de dois pacientes com grandes adenomas hepáticos submetidos ao tratamento minimamente invasivo por meio de embolização arterial. O primeiro caso foi submetido à embolização eletiva, por apresentar múltiplos adenomas, além de hemorragia recente de um desses nódulos. O segundo, caracterizado por vítima de trauma abdominal fechado e rotura de adenoma hepático, foi realizado em caráter de urgência, tendo a paciente sinais clínicos de choque hemodinâmico secundário à hemorragia intra-abdominal. O desenvolvimento de terapias minimamente invasivas locorregionais, como a embolização arterial, trouxe um novo horizonte para pacientes com adenomas hepáticos. Na ressecção emergencial de um adenoma hepático roto, as taxas de mortalidade são de 5 a 10%, enquanto que a ressecção eletiva reduz a 1% esse desfecho. A embolização arterial dos adenomas hepáticos na vigência de hemorragia é tema de debate. Essa constatação aponta para um possível papel da embolização transarterial desses tumores rotos e não rotos, visto que tal conduta poderá limitar a indicação cirúrgica em casos selecionados, resultando na redução da morbimortalidade. O seguimento das pacientes tratadas foi realizado por meio de ressonância magnética e, após 30 dias, já havia diminuição das lesões embolizadas bem como a presença de significativo componente avascular. O controle radiológico, após 12 meses, mostrou ausência de novas lesões e diminuição daquelas embolizadas.

## INTRODUCTION

The recent increase in the number of diagnosed solid liver lesions has been attributed to advances and wider availability of radiological testing^(1)^.

Hepatocellular adenoma is a rare benign solid liver tumor of epithelial origin that primarily affects women of reproductive age. The prevalence of this adenoma increased remarkably in the 1980s and 1990s, which was attributed to the widespread use of oral hormonal contraceptives^(2,3)^. The incidence of hepatocellular carcinoma is 1 to 3/100,000 inhabitants/year in the general population according to current estimates^(4)^.

We report two patients with large hepatic adenomas who were subjected to minimally invasive treatment using selective arterial embolization of the lesions. Elective embolization was indicated in one case due to the presence of multiple adenomas and a recent history of bleeding in one of the nodules. The other case was a victim of blunt abdominal trauma associated with the rupture of a previously unknown hepatic adenoma.

## CASE 1

The first case was a 30-year-old female with a history of obesity and oral hormonal contraception use. She complained of sudden abdominal pain in the right hypochondrium without signs of peritoneal irritation in January 2011.

Laboratory testing showed a reduced hemoglobin level and negative hepatitis B and C serology. Magnetic resonance imaging of the abdomen showed multiple solid, nodular, hypervascular lesions primarily in the right liver lobe, and a dense, heterogeneous, 10.1 × 6.2cm structure in the caudate lobe, providing interface between the right and left liver lobes, compressing the inferior vena cava, dislocating the middle and right hepatic veins and the portal branches of the right lobe. The features of this lesion were compatible with intraparenchymal hematoma related to a bleeding hepatic adenoma.

**Figure 1:**
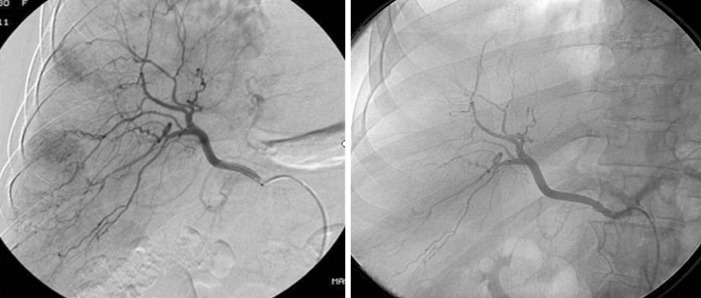
Angiography of the liver before and after embolization. Hypervascular lesions compatible with hepatic adenomas were observed in the liver right lobe, and these lesions are absent in the angiography after embolization

The patient remained asymptomatic during the outpatient follow-up period, but magnetic resonance imaging three months later revealed multiple hypervascular nodules scattered across both liver lobes, suggestive of new adenomas. Digital angiography of the liver confirmed the presence of multiple hypervascular lesions in the right liver lobes. Selective arterial embolization of the largest lesions in the right liver lobe was performed with 300- to 500-micron calibrated microspheres due to the high risk of bleeding and rupture (Figure 1).

**Figure 2:**
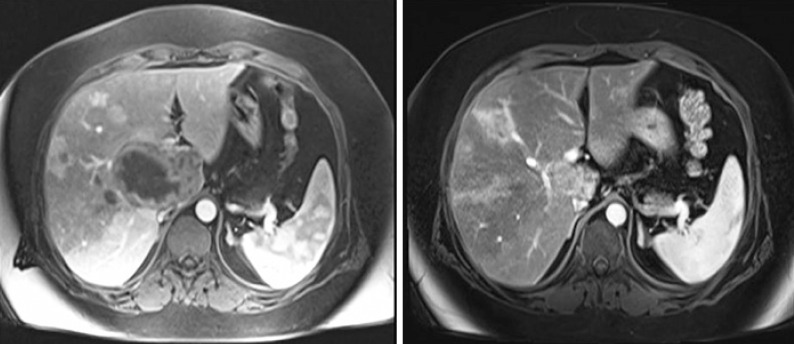
Magnetic resonance imaging before embolization and one year later. Hypervascular lesions in the liver right lobe. The largest one is located in the caudate lobe with central necrosis and external compression of the inferior vena cava. The control magnetic resonance imaging exam performed one year after embolization shows a reduced hypervascular lesion in the caudate lobe, no external compression of the inferior vena cava, and no growth of the remaining hypervascular lesions

Magnetic resonance imaging 30 days after embolization showed reduced embolized lesions and a significant avascular component. No new liver lesions were found on radiological images 12 months later, and the embolized lesions were smaller (Figure 2).

## CASE 2

Case two was a 23-year-old female using oral hormonal contraception who suffered blunt abdominal trauma after falling two meters and hitting the right hypochondrium against a table. She complained of severe abdominal pain and exhibited signs of peritoneal irritation, skin and mucous membrane pallor, and hemodynamic shock.

Laboratory testing showed a reduced hemoglobin level. Initial care in the emergency department, including fluid resuscitation and analgesia, controlled the hemodynamic shock. Computed tomography of the abdomen revealed the presence of multiple solid, hypervascular, nodular lesions, and liquefied hypoattenuating lesions mostly in segments VI and VII. The remainder of the approximately 10 lesions were primarily located in the liver right lobe, and the largest lesion was in segment II/III. The test also identified a large amount of free fluid beside the inferior liver margin and right paracolic gutter that extended to the pelvis. The lower margin of the liver lesions was slightly irregular, which suggested active bleeding into the peritoneum.

**Figure 3:**
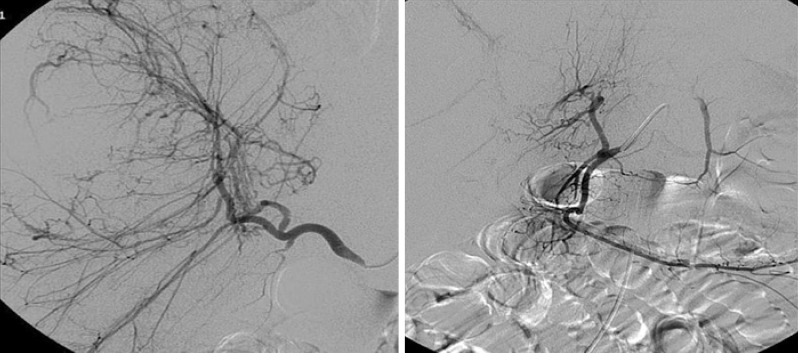
Angiography of the liver before and after embolization. A large hypervascular lesion compatible with hepatic adenoma was observed in the liver right lobe, and the amputation of the intrahepatic arterial branches in the control exam after embolization is shown

**Figure 4:**
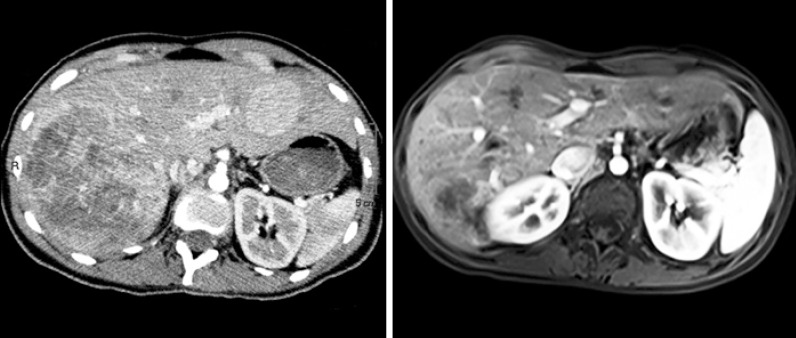
Tomography of the abdomen before embolization and control magnetic resonance imaging one year later. A large hypervascular lesion is observed in the right liver lobe with a heterogeneous aspect suggestive of local hematoma, hypervascular lesions in the left liver lobe, and free fluid in the peritoneal cavity suggestive of abdominal hemorrhage. The magnetic resonance imaging exam one year later showed a significant reduction of the embolized lesions and the absence of new lesions

The patient was transferred to the interventional radiology unit, and digital angiography of the liver was performed to confirm the presence of multiple hypervascular liver lesions. Selective arterial embolization of the largest lesions in the right lobe was performed with 300- to 500-micron calibrated microspheres (Figure 3). The hemorrhage was stopped, and the hemodynamic conditions improved gradually. No new liver lesions were found on radiological images 12 months later, and the embolized lesions were smaller (Figure 4).

## DISCUSSION

The present article reports two cases of hepatic adenomas that were treated using arterial embolization under two different clinical conditions. Arterial embolization in the first case was an elective treatment following an episode of self-limited, spontaneous hemorrhage. The second case was an emergency treatment due to the hemodynamic instability of a patient with bleeding adenoma following abdominal trauma. The therapy was successful in both cases. Bleeding did not relapse during the 12 month follow-up period, and the size of the treated lesions was substantially reduced.

The development of minimally invasive locoregional treatments for the liver, such as arterial embolization and radiofrequency ablation, introduced new approaches for the management of individuals with non-ruptured hepatic adenomas^(5)^. However, individuals in whom hepatic adenomas negatively impact their quality of life or who exhibit signs of malignant conversion on imaging tests should be indicated for tumor resection.

The mortality rates for the emergency resection of ruptured hepatic adenomas vary from 5 to 10%, but these rates are reduced to approximately 1% when resection is elective^(6,7)^. The indication for arterial embolization of hepatic adenomas in the presence of active bleeding is the subject of recent debate in the literature. The reduced mortality rate suggests a role for the transarterial embolization of tumors in the management of ruptured and non-ruptured hepatic adenomas. Follow-up examinations after embolization support the usefulness of this conservative treatment, which might reduce the indication for surgery in selected cases and decrease the morbidity and mortality. The exceptional nature of the treatment of the bleeding hepatic tumor elicited by blunt abdominal trauma further supports the usefulness of arterial embolization under different clinical conditions.

Patients with hepatic adenomas should discontinue oral contraception because these tumors regress after these agents are interrupted^(8,9)^. Contrast imaging studies, such as tomography and magnetic resonance imaging, are crucial components of the patients' follow-up examinations^(10)^.

In conclusion, transarterial embolization is an acceptable treatment for hepatic adenomas independent of the presence or absence of bleeding. The use of this therapy facilitates hemodynamic control in cases with bleeding and reduces tumor size. These findings should be confirmed in prospective multicenter studies.
